# The Triggering Mechanism of Short Video Customer Inspiration – Qualitative Analysis Based on the Repertory Grid Technique

**DOI:** 10.3389/fpsyg.2021.791567

**Published:** 2021-12-09

**Authors:** Peng Gao, Heng Jiang, Ying Xie, Yu Cheng

**Affiliations:** ^1^School of Economics and Management, Northwest University, Xi’an, China; ^2^School of Foreign Languages, Northwest University, Xi’an, China

**Keywords:** short video ads, customer inspiration, cognitive processing, emotional process, repertory grid

## Abstract

It is believed that stimulating the inspiration of short video consumers might be an effective way to attract and maintain the attention of consumers so that they are willing to respond positively to short video ads. Therefore, in order to explore the source of customer inspiration in short video and its cognitive psychological process, the text and grid data collected from an interview among 25 short video users have been qualitatively analyzed by Kelly Grid Technology in order to construct the formation path model of short video customer inspiration, and find out its source, triggering mechanism, and influencing factors. It is found that the inspiring informational content characteristics include richness, reliability, vividness, and fluency of emotional content characteristics, fun, novelty, and narrative. However, the characteristics of commercial content in short video ads hinder the inspiration of consumers. The study also reveals that an internal mechanism of inspiration stimulation is built on some cognitive processes (i.e., presence, processing fluency, perceived innovation, perceived convenience) generated by informational content, and emotional responses by emotional content (i.e., curiosity, surprise, enjoyment, etc.). In addition, it is shown that personal involvement enhances the relationship between the inspiring content characteristics and consumer inspiration. As a result, customer inspiration and engagement in short video ads are highly enriched. Findings provide implications for short video platforms and online marketers.

## Introduction

With the explosion of information in the era of social media, the limited concentration of consumers is easily distracted or even hardly captured. Despite the vitality and abundance of internet video advertisements, they are also short of effective method to attract the attention of consumers (Teixeira et al., 2012). Therefore, to better engage and maintain the attention of viewers in communication through digital channels, online content producers begin to create short video advertisements (featured by its limited length within 30, 20, or even 10 s) to promote the dissemination of product information and consumer purchase (Liu et al., 2018). A derivative of the Internet age, short video differs from the tradition long video in that filming and editing of a seconds-long video can be completed on mobile intelligent terminals and shared in real-time on social media platforms. Short video ads, therefore, have become a direct pathway to achieve commercial value of short video platforms such as TikTok and Kwai by raising the transmission and acceptance of short video ads among users who have been gathered in a huge number and precisely directed to certain personalized advertisements. Undoubtedly, when short video becomes one of the most popular recreational forms in the mobile Internet era, short video ads are naturally transformed into a major tool for brands and merchants to promote new products and establish communication and positive relationships with their customers.

While short video ads can be an effective marketing tool, it is far from enough to simply rely on the unique or appealing content to improve its marketing performance. On the one hand, consumers who have been constantly suffering from concentration difficulties may swipe the phone quickly to avoid watching short video ads because of their aversion to the commercials (Nikolinakou and King, 2018). Additionally, given the low purchase conversion rate of short video ads, whether the content that attracts the attention of consumers can motivate purchasing behavior remains an important challenge for current research. For these reasons, the critical issue that should be addressed by online marketers is to find out effective ways to attract and stimulate positive response from consumers. The relevant studies on consumer behavior are, therefore, highly announced and promoted. However, few studies have been found to concern about changes and influencing factors in consumer behavior in relevant context. Although Liu et al. (2018) found that effective editing of short video scenes can enhance the willingness of consumers to watch the video. The findings of which are more applicable to the edition of old videos produced by brand owners, rather than to the creation of new ads, both showing significant difference (Diwanji and Cortese, 2020). Furthermore, prior studies have mainly explored the content characteristics that drive online behaviors such as browsing and sharing in the context of video advertising (Teixeira et al., 2012; Yang and Wang, 2015; Lee and Hong, 2016; Akpinar and Berger, 2017; Nikolinakou and King, 2018; Tellis et al., 2019; Guitart and Stremersch, 2021). It is still questionable whether the theoretical framework based on traditional video ads can be transplanted to the study of short video ads that are fundamentally different in terms of generating and propagating information from the former. For example, TV ads are accused of high expense and low consumer engagement, and online long video ads fail to fit the “staccato” quality of online browsing. Since existing research have difficulty in the solution of effective conversion of consumer attention into real purchase, further research should be developed with a focus on short video advertisements.

Recent research on “customer inspiration” may shed light on the way of capturing and maintaining the attention of short video audiences so as to inspire their positive response. Customer inspiration consists of cognitive activation and intention driver, which describes the internal activation state that encourages the attention of the consumer and new ideas for practice (Thrash and Elliot, 2003, 2004; Böttger et al., 2017). Specifically, customer inspiration will lead to positive emotional experience such as pleasure and surprise. This is an important way to arouse and maintain the attention of the consumer and improve the efficiency of video advertising communication (Teixeira et al., 2012). Customer inspiration will also lead to goal pursuit motivation. Consumers in an inspired state will be motivated to achieve consumption-related goals so that they will impulsively buy some products or services beyond their plan (Böttger et al., 2017). Clearly, inspiration not only encourages the active participation of the consumer in short video ads and voluntary search of inspirational content, but also motivates purchases of products and services within their targets. According to some recent studies, social media, as a new marketing channel, has become an important source of inspiration (Dale et al., 2017; Sheng et al., 2020). Creative short videos should have unique advantages in triggering the inspiration of customers, but no study has been reported to specifically explore the source of the inspiration of customers and its psychological processes. Therefore, this article attempts to address this gap in terms of customer inspiration and relevant theories. Specifically, we aim to answer the following research questions: (1) What are the characteristics of short videos that help to trigger customer inspiration? (2) What is the psychological mechanism of customer inspiration triggered by short video? (3) What are the influencing factors on the triggering mechanism? Resolutions to these problems will enrich the understanding of customer inspiration in short video advertising and help to improve the purchase conversion rate of short video advertising.

## Literature Review

### Characteristics of Advertising Content

In spite of the significant differences between video advertising and short video advertising in terms of production methods and communication forms, they both essentially reconstruct product attributes, functions, and other business information through audio-visual symbols. In addition, considering that short video research is in the ascendant, it is advisable to refer to the previous studies on video advertising so as to explore the advertising features that can promote communication and consumer purchase. According to the content feature framework of online video advertising shared by consumers (Tellis et al., 2019), informational content, emotional content, and commercial content are the most important driving factors. These factors are controlled by advertisers and have been tested true both theoretically and empirically.

In the classic integrative models of advertising, informational route and emotional route are two main paths to influence consumer attitudes (Macinnis and Jaworski, 1989). They can have a differentiated impact on searching, sharing, rating, and purchasing (Yoo and MacInnis, 2005; Lee and Hong, 2016; Akpinar and Berger, 2017; Tellis et al., 2019; Guitart and Stremersch, 2021). First, information focused content aims to convey information cues related to product attributes, price information, promotional activities, and brand events (Chandrasekaran et al., 2017), which usually involves a propagating argument or factual description (Tellis et al., 2019; Guitart and Stremersch, 2021). Obviously, by objectively describing the advantages of the brand or product, the informational content of advertisement satisfies the appeal of consumers and improves brand beliefs (Chandy et al., 2001). Some scholars also believe that informational content is crucial in persuasion and communication (Hsieh et al., 2012). Consumers will regard this kind of information as fairer and less manipulative, and the positive inference will increase brand search and purchase intention (Chandrasekaran et al., 2017). However, Tellis et al. (2019) takes the fact-centered informational content as particularly dull and boring, and even irritating unless in such high-risk situations that involves new products or high prices, which results in lower advertising sharing willingness. In response to the above contradictory conclusion, Akpinar and Berger (2017) proposed that if consumers are motivated to actively deal with advertising information, they are to obtain brand and product knowledge that help them to make purchasing decisions, and ultimately improve their evaluation of informational content. Therefore, the informational content of advertisements provides consumers access to awareness of useful or unique features of the product in some special situations (Yoo and MacInnis, 2005), and, as a result, promotes positive consumer attitudes and behaviors.

Secondly, emotion-focused content aims at arousing the emotional response of consumers (Chandrasekaran et al., 2017), which includes positive or negative emotions (Tellis et al., 2019). Different from the complex impact of informative content on consumer response, advertisements with emotional appeal are believed to be more persuasive (Berger and Milkman, 2012; Teixeira et al., 2012; Nelson-Field et al., 2013; Nikolinakou and King, 2018). In fact, emotional content produces many important advertising results, such as viewing time (Teixeira et al., 2012) and advertising sharing (Akpinar and Berger, 2017; Tellis et al., 2019), purchase intention (Lee and Hong, 2016), and sales (Chandy et al., 2001; Guitart and Stremersch, 2021). Furthermore, previous literature explores the advertising characteristics that stimulate the emotional experience of consumers. For example, Tellis et al. (2019) found that drama, plot, characters, and surprise are positive factors that trigger emotions and sharing activities, while mood and music can build emotions and orientations (Yoo and MacInnis, 2005). More importantly, Liu et al. (2018) found that effective editing of rhythm, sequence, and sound in a short video can control the emotional experience of consumers and improve their willingness to watch. Generally speaking, except under some risky conditions, the emotional characteristics displayed in the ads will produce a greater impact on consumer behavior.

In addition, distinct from the content produced by non-marketers, commercial nature and persuasive intention are important characteristics of advertising. Tellis et al. (2019) defined commercial content as the one that influence consumer support to branded product or service, such as a brand logo on an advertising screen. It is worth noting that, the persuasive knowledge activated when the advertisers use commercial content to establish positive brand association for the consumers will also attract the attention of consumers to the features of implanted products, which in return results in the undervaluation or resistance of consumers to persuasive information (Friestad and Wright, 1994), and even unwillingness to share (Akpinar and Berger, 2017). Worse still, the significant commercial motivation of video advertising reduces the effectiveness of content characteristics that originally tend to promote the willingness of consumers to share (Tellis et al., 2019). In summary, the higher the intention of video advertising, the lower the willingness of consumers to share.

### Customer Inspiration

Customer inspiration is the specific application of the classical social psychology concept “inspiration” in the marketing situation (Thrash and Elliot, 2003, 2004), i.e., a temporary activation state that prompts the customer to move from a marketing-guided mindset to an internal pursuit of consumption-related goals (Böttger et al., 2017). In this article, the concept of “short video customer inspiration” is used as the specific representation of customer inspiration in the context of short video advertising. Further, Böttger et al. (2017) proposed that customer inspiration is a second-order construct consisting of ‘inspired-by’ and ‘inspired-to’ states. The inspired-by activation state relates to the reception of a marketing-induced new idea and the shift in customer awareness toward new possibilities. Comparatively, the inspired-to state relates to the intrinsic pursuit of new ideas and consumption-related goals. Many studies have confirmed that concerns about customer inspiration are valuable in predicting such positive behavior, emotion, and attitude as increased purchasing intention and purchasing behavior (Winterich et al., 2019), pleasure and surprise (Böttger et al., 2017), and customer satisfaction and loyalty (Herhausen et al., 2019).

By combing relevant literature, a large number of empirical analyses have been conducted on the sources of customer inspiration, and their results provide certain implications for research on the triggering mechanism of customer inspiration in short videos. Customer inspiration is not spontaneous but induced by external stimuli of intrinsic value (Thrash and Elliot, 2004). Böttger et al. (2017) proposed that the most inspirational marketing stimulus contains three source features, namely, the provision of inspirational content, appeals to use the imagination, and elicitation of an approach motivation. It is found in some empirical studies that utilitarian and hedonic content have a positive impact on customer inspiration (Izogo and Mpinganjira, 2020). For example, novel and vivid advertising information helps consumers acquire new ideas and imagination, thus triggering customer inspiration (Böttger et al., 2017; Winterich et al., 2019). In addition, advertising information targeted to the specific emotional demands of consumers can also be inspiring. Bischof et al. (2020) observed that the desire of consumers for exploration is contented by the astonishing subscription service, and they are therefore inspired to try new things. Similarly, donation advertisements that evoke strength emotion (Liang et al., 2016) and travel destinations that lead to attachment emotion (Khoi et al., 2019) also have unique enlightenment because they can arouse the inner interest and motivation of consumers to pursue their goals.

### Summary of Literature

From the above literature review, it can be seen that: (1) previous studies on advertising are based on the conceptual framework of informative, emotional, and commercial content, deeply exploring the factors that influence the effectiveness of advertising. Most of the discussions focus on advertising attitude, brand evaluation, viewing behavior, purchase intention, sharing intention, sales, and other dependent variables. However, as mentioned above, customer inspiration that includes cognitive activation and intention-driven components is more helpful to resolve the urgent problems in short video advertising, that is, how to attract and maintain the attention of consumers and prompt them to make a positive response. Unfortunately, there is no research taking customer inspiration as the key concept to explore the effectiveness of short video advertising, which should have been a new perspective in the field. (2) A number of advertising features related to information, emotion, and commerce have been identified, and marketing drivers with heuristic traits (informative and emotional) have also been extensively validated. However, the short video, as a new media, is different from other social media applications in terms of content generation, display, browsing and transmission (such as graphic posts, long videos, micro-films, and live broadcasts). Moreover, customer inspiration largely depends on the situation that generates it. The existing characteristics of inspiration may not be applicable to short video advertising. It is therefore necessary to use the current content framework to explore new factors that influence the generation of inspiration of short video customers and its triggering mechanism.

## Methodology

### Repertory Grid Technique

This study aims to explore the triggering mechanism of customer inspiration in short videos. In view of the lack of mature theories and studies on this issue, the qualitative research method is given priority. Specifically, the Repertory Grid Technique (RGT) has been adopted in research interviews and data collection. RGT originates from the personal construct theory in cognitive psychology. The constructs accumulated in life are used to explain and predict the events around (Kelly, 1955). Kelly (1955) pointed out that an individual only has a limited number of constructs that are bipolar in nature. For example, the description of consumers of short video advertisements can be ‘interesting or boring,’ ‘novel or plain,’ ‘lively or bald,’ etc. Therefore, RGT is mainly used to extract individual constructs that judge complex things or phenomena (Kelly, 1963). This is especially appropriate for exploring the topics whose answers are indirectly known and implicit knowledge that cannot be conveyed directly (Goffin et al., 2006).

Although customer inspiration is not essentially a mystic experience (Thrash and Elliot, 2004), it is still challenging for consumers to specify the concept of or access to inspiration (Rauschnabel et al., 2019). In addition, compared to other qualitative methods that are easily affected by subjective bias of researchers in data collection, RGT grants respondents the maximum freedom to comment on a topic so as to ensure the authenticity and accuracy of the data (Goffin, 2002), and therefore has been gradually used as an effective qualitative instrument in marketing research (Lemke et al., 2011; Macdonald et al., 2016; Kawaf, 2017). Recent studies also showed that RGT is particularly applicable for the study of the cognitive and emotional experience of digital consumers (Kawaf and Istanbulluoglu, 2019). It is worth mentioning that some novel methods similar to RGT (such as Dynamic EMCUD and VODKA) have recently been developed to obtain dynamic knowledge that is constantly updated and evolving with environmental changes (Lin et al., 2008; Tseng and Lin, 2009). Other methods, like ZMET, were used to resolve important problems in marketing practice by integrating RGT in the method (Coulter et al., 2001). Obviously, in these methods personal construct theory and RGT serve as crucial access to consumers tacit knowledge, which also confirms that RGT allows a better solution to problems on cognitive decision making. In general, RGT helps to uncover the source and cognitive process of inspiration when consumers are exposed to short video advertising.

### Sample Selection

As a unique qualitative research method, RGT is characterized by numerous and time-consuming interview steps, which makes it possible to extract enough constructs from 15 to 25 samples to meet the research needs (Tan and Hunter, 2002). With a theoretical sampling method, the interviewees in this study are selected on the basis of judgments of the researchers and research questions. Specifically, the respondents must have high intensity both in browsing short videos and of purchasing relevant products or services on the short video platform. According to the statistical report of “2019 White Paper on Marketing Strategy of China’s Short Video Enterprises” released by iResearch, the proportion of male and female users of the short video applications of China in 2019 is close to 1:1 and the user group is mainly under the age of 24. Therefore, this study also balances the gender, age, and other characteristics of respondents according to the portrait characteristics of current short video users. In this study, 25 short video users were interviewed (female 48%, *M*_age_ = 22.4). The interview was conducted face-to-face, with each interview lasting from 33 to 97 min (*M* = 56) (Table 1). Ultimately, the requirement of theoretical saturation has been reached, that there were no other new ideas from the respondents.

**TABLE 1:** Information of respondents.

No.	Gender	Age	Browsing frequency/week	Purchasing frequency/Month	Duration of interview/min
1	F	25	4	2	40
2	M	23	7	3	38
3	F	26	3	1	53
4	M	21	8	3	47
5	F	19	6	4	35
6	M	24	12	3	51
7	M	19	7	2	57
8	M	20	5	1	36
9	M	25	9	2	45
10	F	27	10	4	33
11	F	28	8	1	60
12	F	19	6	2	49
13	F	18	7	3	46
14	M	22	20	2	73
15	M	19	14	2	69
16	F	23	10	3	77
17	F	23	8	1	66
18	M	25	7	2	64
19	F	20	12	4	71
20	M	20	9	2	40
21	F	20	7	2	52
22	M	25	10	3	68
23	M	24	21	3	84
24	F	19	6	2	52
25	M	26	15	3	97

### Data Collection

In the formal interview, researchers first explained the meaning of customer inspiration to the respondents to ensure that they can understand the concept properly. The respondents were informed of the two parts in the experience of customer inspiration, namely, to obtain new ideas or possibilities from the external marketing stimuli, thus experiencing a ‘flashing’ moment of being enlightened as shown in the words like “Aha!,” “Eureka!,” etc., and to generate motivation to implement new ideas, such as the strong desire to buy or use products. The specific interview steps are as follows. First of all, each respondent needs to watch the 16 highly inspiring short video advertisements (elements)^1^ prepared by the researchers one by one, and to select from them three ads that can stimulate inspiration and three that cannot. The selected six ads are written on a separate card in digital form. Secondly, according to the highly respected triads method (Kelly, 1963), three cards with short video names are randomly presented to the respondents who are then required to answer the following question: “In terms of your choice, which two of the three short video ads are more similar and different from the third one? And what are the similarities?”. Answers to this question will lead to a bipolar construct, the positive construct being recorded on the right side of a prepared square and entitled as a novel construct, while the negative one on the left side as a plain one. Next, in order to establish links between elements and constructs, after discussing the specific meaning of the first construct, the respondents need to evaluate all the six short video advertisements selected before with a Likert 7-point scale. In this study, a Likert 7-point scale was adopted because it could give the respondents more freedom to rank elements (Tan and Hunter, 2002). It is worth noting that the constructs extracted by the triple method are more concrete, thus differentiating from the inspiration that is more abstract. To this end, laddering interview technique is applied to elicit higher-order, more abstract constructs that are closer to the target in the personal construct system (Reynolds and Gutman, 1988). In particular, after the respondents answering the initial questions, a series of laddering questions will be raised to establish higher-level constructs, like “Why is this important to you?,” “What does that mean to you?,” “What do you think about it?”. So far, an integrated triple discussion has been completed. A complete grid example is shown in Table 3, consisting of elements, construct, and connection (Easterby-Smith, 1980). The process where the constructs have been formulated with the method of triads and laddering is repeatedly applied with the new cards until the respondent fails to produce any new construct. During the whole experiment, no construct that has been mentioned in the previous step is allowed to be repeated. The possibility of creating new construct from each triad encourages the respondents to think deeply about the connotation of customer inspiration in the short video. Reger (1990) disagreed with the necessity of repeating all the triads, for in most of the cases 7–10 groups are enough to exhaust all the constructs of the interviewee. The number of triads in this study ranges from 5 to 13 (*M* = 8.44, *SD* = 1.64), which confirms the above conclusions. In the square example of Table 3, the first respondent produced five constructs.

**TABLE 2:** Inspiring short video ads.

Product	URL	Length/second	Volume of likes	Volume of comments	Volume of forwarding
Disposable sponge mop	https://v.douyin.com/sfVkYj/	15	136w	2.2w	8.4w
Shadow lamp	https://v.douyin.com/sfV8JR/	15	142.4w	1.2w	1.1w
Collocation of clothes	https://v.douyin.com/RebuF5x/	13	113.2w	4.1w	7.3w
Night table	https://v.douyin.com/sfpgLf/	20	74.1w	1.2w	2.9w
Glass	https://v.douyin.com/sftg9b/	13	187.8w	5.5w	11.6w
Coke inversor	https://v.douyin.com/sfchvQ/	25	189.2w	4.8w	3.1w
Engraving machine	https://v.douyin.com/sfnsxH/	24	60.2w	1.8w	1w
Porphyra rice	https://v.douyin.com/sf4XqU/	25	164.6w	3.0w	6.2w

**TABLE 3:** Samples of grids.

Negative construct pole (1)	Short video ads (Elements)						Positive construct pole (7)
	Short video 1	Short video 2	Short video 3	Short video 4	Short video 5	Short video 6	
(1) Plain	5	1	1	7	1	7	Novel
(2) Fail to arouse curiosity	6	1	1	7	1	6	To arouse curiosity
(3) Explicit promotion purpose	6	4	1	2	1	2	Implicit promotion purpose
(4) Irrelevant to personal needs	6	2	1	7	1	5	Relevant to personal needs
(5) Uninspiring	6	1	1	7	1	6	Inspiring

*Number 1–5 in each line represents the construct formulated by the respondents. The constructs of all the six short video ads are evaluated in accordance with numbers 1–7, in which 1 represents the negative construct pole (uninspiring), while 7 the positive construct pole (inspiring).*

## Data Analysis

The recorded audio interview process was transcribed into a written text of more than 60,000 words before data analysis. The analysis of quantitative and qualitative data of transcribed text precisely follows the method adopted by Goffin et al. (2006), which includes three steps: standardization of construct names, categorization of constructs, and identification of key constructs.

### Standardization of Construct Names

A small number of constructs in the interview are same in meaning but slightly different in expression. After carefully examining all the text content and grids, two researchers in charge of data collection identified and standardized the duplicate constructs. For example, 15 interviewees admitted that they are inspired when the short video ads provided a sensory experience of audio-visual conformity. But they used ‘audio-visual conformity,’ ‘audio-visual coordination,’ ‘audio-visual match,’ ‘audio-visual fit,’ and other expressions in the positive construct pole. The inconsistency of this kind was also found in the negative construct pole. To the end, ‘audio-visual conformity’ was determined as the standard construct name. Through such a process of standardization, 279 initial constructs have been simplified to 50. Furthermore, in order to ensure the significance of the analyzed constructs, the constructs that had been mentioned by at least three interviewees (≥12%) were finally retained, with a total of 39 constructs.

### Categorization of Constructs

Based on the inherent meaning of and interaction between constructs, 39 standardized constructs were categorized in accordance with their themes. This categorization strictly followed the four steps recommended by the prior research (Goffin and Koners, 2011), namely, identifying categories, allocating constructs to those categories, tabulating the results, and establishing the reliability of the category system. In the first step, each of the 39 constructs was written on a separate card, including its name, polar position, and typical description of the construct. Next, the preliminary categories composed of names, definitions, and construct distributions were established by discussion between two researchers who were in charge of data collection. At the same time, an independent researcher was invited to classify these cards, and the consistency of the two classification results was calculated accordingly. The reliability index between the coders was 74.36%, which was obtained by dividing the number of the constructs distributed in one category by the total number of constructs. Although higher than the critical level of 70%, it is advisable to modify the definition and distribution of categories to remove ambiguity. Therefore, the second independent researcher classified the constructs based on the modified names and definitions, and got the reliability index of 84.62%, which was much higher than 70%. With the reliable result, 39 constructs were eventually allocated into 18 categories (Table 4).

**TABLE 4:** Categorization of constructs and identification of key constructs.

Category	#	Concept name	Frequency (%)	ANV (%)	Key constructs
Richness: the ability short video ads to carry data, i.e., to meet consumers’ need for quantity of information and to reduce ambiguity.		Richness in scenario	6(24)	10.24	False
		Richness in information	6(24)	7.27	False
Reliability: The authenticity and credibility of short video ads.		Authenticity of content	8(32)	8.21	False
		Professionality of content	6(24)	10.18	False
Vividness: Short video ads enable consumers to create a clear picture of the products, concepts, and situation in their minds.		Display of details	3(12)	5.45	False
		Multi-sensory experience	3(12)	10.58	False
Fluency: Short video ads contains an audio-visual experience that makes it easy for consumers to perceive and identify product features.		Audio-visual conformity	15(60)	5.80	False
		Smooth rhythm	8(32)	7.76	False
		Visual impact	5(20)	9.40	False
		Visual aesthetics	4(16)	11.70	False
		Suitable scenario	3(12)	9.79	False
		Visual fluency	3(12)	10.28	False
Fun: enjoyable, interactive, funny, humorous, and comical short video ads.		Fun	4(16)	9.92	False
Novelty: fresh, surprising, creative, and unique short video ads that produce different experience.		Novelty	15(60)	10.40	True
Narrativity: short video ads that describes outcome in the form of ‘storytelling’.		Display of plot	6(24)	8.13	False
		Making of Surprise	4(16)	9.21	False
		Narratives of story	4(16)	0.87	False
Commercial intention: short videos contain a clear marketing label with biased and persuasive intention.		Marketing purpose	10(40)	9.82	True
Presence: consumers perceive the authentic situation by virtual environment.		Immersiveness	9(36)	2.15	False
Processing fluency: subjective perception of the ease with which consumers process information.		Clarity of the topic	6(24)	8.95	False
		Comprehensibility	4(16)	3.99	False
Perceptual innovation: consumers’ subjective judgments of novelty in short video ads.		Perceived difference	6(24)	11.56	False
		Perceived novelty	3(12)	10.83	False
		Perceived creativity	3(12)	7.35	False
Perceptual convenience: consumers’ perception of practicality and convenience of short video ads.		Perceived influence	11(44)	9.68	True
		Perceived practicality	10(40)	8.26	False
		Perceived convenience	5(20)	9.87	False
Curiosity: the desire to acquire missing information, i.e., consumers’ awareness of the “information gap” between what they currently know and what they want to know.		Curiosity	14(56)	11.28	True
Surprise: differences derived from consumers’ psychological schema, i.e., perception of the unexpectedness of short video ads.		Surprise	4(16)	10.96	False
Enjoyment: consumers have a pleasant and happy feeling when watching short video ads.		Enjoyment	4(16)	6.48	False
Conceptual inspiration: Consumers are receptive to new ideas and possibilities promoted in short video ads.		Divergent thinking	36	10.15	True
		Cognition challenge	6(24)	10.29	False
		Association initiation	6(24)	10.67	False
		Interest stimulation	4(16)	10.14	False
		Knowledge acquisition	3(12)	7.4	False
Behavioral inspiration: consumers develop intrinsic incentives to achieve consumption-related goals.		Purchase motive	8(32)	12.52	True
		experiential motive	7(28)	10.72	True
Personal involvement: consumers perceive the relevance of short video ads based on their internal needs, values and interests.		Relevance in interest	12(48)	10.94	True
		Relevance in demand	11(44)	9.76	True

### Identification of Key Constructs

Goffin et al. (2006) proposed that frequency and variability are important indicators to the establishment of key constructs. Firstly, the frequency indicator is satisfied when the construct is articulated by at least 25% of respondents. In this part, 14 important constructs met the frequency threshold. Secondly, the variability indicator is used to quantify the relative importance of a construct due to the significant difference in the evaluation of construct, and thus effective in distinguishing highly inspiring short video ads from low inspiring ones. Specifically, the variability of a construct is a measure of the scale of ratings compared to all the other constructs. The higher the variability of a construct is, the greater is its importance to the respondent. The variability of a given construct represents its contribution to the total variance. Additionally, since the measurement of variability depends on the number of constructs contained in each independent square, the variation value of each construct needs to be standardized. In this study, Average Normalized Variability (ANV) of constructs was 8.96%. When the ANV of a construct is greater than this threshold, this construct can be used to distinguish different types of short video ads, i.e., whether they inspire customers. In all, 25 important constructs were found to meet the requirements of the variability threshold. Furthermore, a key construct is defined as the one to satisfy both the threshold of frequency and variability. Accordingly, nine key constructs were identified as the most convincing criterion to distinguish short video ads with varying degrees of inspiration.

## Results and Analysis

**FIGURE 1:**
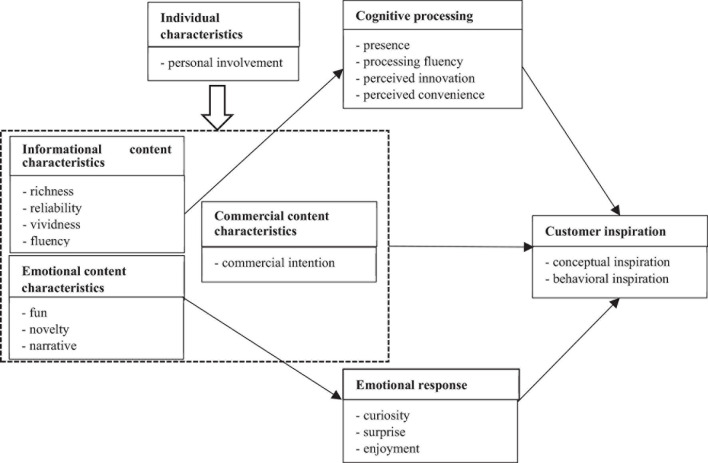
Formation path model of short video customer inspiration.

As shown in Table 4, the constructs derived from RGT are more extensive. Some constructs are related to the informational content characteristics (e.g., richness in scenario, authenticity of content, audio-visual conformity) and emotional content characteristics (e.g., fun, novelty, surprise) of short video ads. Some are likely to be created by cognitive processing (e.g., immersiveness, clarity of the topic, and perceived difference) and emotional response (e.g., curiosity, surprise, and enjoyment) of consumers when they are exposed to short video ads. Some others are affected by the personal characters of consumers (e.g., relevance in interest and demand). In addition, the respondents brought up some constructs connected to the connotation of customer inspiration (e.g., divergent thinking and purchase motive). The analysis of grid and text data proved a significant interaction between these constructs. Therefore, it is necessary to establish relation structures on the basis of the construct category and formulate the path model of customer inspiration of short videos (Figure 1).

According to the model, customer inspiration is significantly affected by the inspiring informational content characteristics, emotional content characteristics, and commercial content characteristics in which the characteristics of informational content mainly activate the cognition-dominated processing mechanism, that of emotional content which arouse the emotion-dominated processing mechanism, and of commercial content which exert a direct impact on customer inspiration. Besides, the possibility of triggering inspiration in short videos is influenced by customer personality. This part of the article is to illustrate the source, triggering mechanism and influencing factors of customer inspiration in short videos, and draws the corresponding research propositions.

### Source of Customer Inspiration in Short Videos

#### Informational Content Characteristic

Customer inspiration experience is highly correlated with cognition. The key to trigger an inspiration lies in that arousal cognitive events produce new or better possibilities (Thrash and Elliot, 2004). This is reflected in marketing as informational content that can stimulate the imagination of consumers or broaden their mental horizons (Böttger et al., 2017). According to the results of data analysis, inspiring informational content characteristics include richness, reliability, vividness, and fluency. First, short videos with rich content helps to improve the assessment of consumers to the value of advertising content, prompt better understanding of the multiple impacts of products on daily life, and stimulate inspiration, which can be determined by the quantity of product and scenario information. As observed by a respondent, “*Many scenes in the short videos about laser keyboard and engraving machine have been switched, so I think they have a lot of uses. The scenes of the other two videos are relatively unchanged, nothing new*” (Respondent 11). Secondly, the reliability of short video ads refers to the credibility of the informational content, with an intention of helping consumers better understand the product features. This raises their initiatives to accept the new ideas guided by marketing. Reliability depends on the authenticity and professionality of content. As one respondent puts it, “*The short video of smart suitcase reflects the feelings of both the users and the onlookers. This can be called objectivity. But in the video about cookies and milk, only one person and his experience are recorded. That is too subjective. If a short video fails to display every aspect of a product, I will not pay much attention to it*” (Respondent 06). On the other hand, based on dual coding theory, the interpretation of an individual of informational content varies significantly from linguistic to non-linguistic system (Paivio, 1991). It is found in this study that consumers can be better inspired with vivid and fluent presentation of information when there is no great difference in the informational content. For example, vivid content enhances the imagination of consumers about the product due to the exposure to more specific information and activation of a better sensory experience. Similarly, a clear flow of content in advertisement helps to create a coherent viewing experience and better perception of product features, and therefore impulse consumers to try or purchase the product. This is the result of such constructs as audio-visual conformity, smooth rhythm, visual impact, and other perceptual feelings.

In the above constructs, the vividness and fluency of video content have been proved to attract the attention of consumers and stimulate their imagination (Hsieh et al., 2012). Although Böttger et al. (2017) asserted that the vitality of advertising has a positive impact on customer inspiration, this study reveals different findings, i.e., the richness and reliability are also inspiring, and the related constructs exceed the requirements of frequency and variability threshold. Therefore, these constructs should be taken as important triggering factors of inspiration in the design of short video ads. Accordingly, the following proposition is obtained:

P1: The richness, reliability, vividness, and fluency of short video ads promote the generation of customer inspiration.

#### Emotional Content Characteristic

Different from the informational content characteristic that convince consumers to accept new ideas and possibilities promoted in marketing in the way of persuasion, communication, and knowledge dissemination, the emotional content characteristic of short video ads may generate the intrinsic motivation of consumers to pursue consumption-related goals by stimulating their positive emotional response (Thrash and Elliot, 2003), which is manifested in business environment as a great desire to purchase or use products (Böttger et al., 2017). In short video ads, the characteristics of inspiring emotional content include fun, novelty, and narrativity. First of all, interesting and funny short video ads can attract attention and stimulate surprise and create pleasure so that the intrinsic motivation (hedonic motivation) of consumers is inspired to share interesting advertising content or take actions to buy products. For instance, one respondent emphasized the significance of enjoyment in his answer to the question about two similar inspiring short videos. “*The video of coke inversor is really boring. I didn’t see any humor or laughing point in it. But the other two videos are more interesting. I have an impulse to buy the product and get the pleasure as shown in the videos, or just buy it as a gift for my kids*” (Respondent 10). Secondly, novel short video ads have prominent emotional driving effect because of their distinctive content or the way it is presented. It is found in the study that novelty brings about surprise and curiosity that provide consumers strong desires to explore new things. In addition, narrativity depicts an intense emotional experience created by the story in short videos, which also encourages an incentive to the pursuit of consumption-related goals. Narrativity is composed of three elements, namely, plot, surprise, and story, with its intensity increased by the application of these elements. In fact, respondents can accurately identify the organization of content that drives emotional experience: “*The videos of porphyra rice and shadow lamp adopted a way of story telling. They were shot in a very dramatic way. I just couldn’t take my eyes away*” (Respondent 18).

In accordance with the findings in the previous research about the novelty in customer inspiration (Böttger et al., 2017; Winterich et al., 2019), this study confirms that novelty, as a key construct, can most effectively distinguish between high and low inspiring short video ads. In the literature on video advertising, Berger and Milkman (2012) found that only videos with enjoyable content can attract attention and bring audience pleasing fulfillment. Tellis et al. (2019) confirmed that dramatic elements (such as plot, character, and surprise) in video advertising can arouse the positive emotions of consumers and enhance engagement of advertisements. Different from willingness to share, fun and narrativity also bring insight and inspiration to customers. Based on this, the following proposition is obtained:

P2: Fun, novelty, and narrativity of short video ads promote the generation of customer inspiration.

#### Commercial Content Characteristic

Studies on video advertising have shown that high level commercial content negatively affect sharing behavior (Akpinar and Berger, 2017; Tellis et al., 2019). A similar trend is also found in this research that short video ads with distinctive commercial intentions will stimulate the avoidance motivation of consumers that seriously hinders the generation of customer inspiration. During the interview, the respondents usually swiped the screen of the phone quickly at the first sight when browsing the short video ads with strong commercial intent. One of the respondents pointed out the negative impact of commercial intent on inspiration experience: “*As to the yogurt candy videos, the sales pitch is too purposeful. I just want to skip it. The other two videos are not trying to sell anything, they just tell you what it is*” (Respondent 08).

As previous studies have shown, extrinsic incentives (such as low price and discount) in advertisements can stimulate the avoidance motivation of consumers. But approach motivation will arouse the inspiration of consumers (Böttger et al., 2017). It is also confirmed in this finding that the frequency and variability of marketing purpose become the condition of key construct and produce significant negative effects on the obtainment of inspiration through short video ads. Therefore, the following proposition is obtained:

P3: Commercial intention in short video ads hinders customer inspiration.

### The Formation Path of Short Video Consumer Inspiration

During the interview, the respondents were often found to spontaneously elaborate their preference to a construct pole, which, in many cases, helps to establish a very clear causal path (Lemke et al., 2011). In the absence of spontaneous interpretation, the laddering interview technique also helped to refine the attribution process (Reynolds and Gutman, 1988). The study results reveal that the cognitive processing caused by inspiring informational content characteristic and the emotional response aroused by the emotional content characteristic are the internal mechanisms for the stimulation of customer inspirations in short videos (Figure 1).

#### Cognitive Processing

Because the informational content focuses on rational description, it mainly activates the cognitive dominated processing mechanism (Yoo and MacInnis, 2005), i.e., thoughts and inferences of consumers when they are exposed to advertising (Cacioppo et al., 1981). This study finds that the informational content of short videos promotes the perceived innovation and perceived convenience of consumers, which is greatly contributing to the formation of customer inspiration. These two cognitive processing paths indicate that consumers understand the competitive advantages of the product, express their understanding and appreciation of the advertising informational content, and in turn create a positive, clear feeling of self-improvement. For example, some respondents perceived the innovative value of short video ads and had an aha experience by saying “*Wow! It can be used like this!*” (Respondent 05). Social psychology shares agreement in that inspiration is closely related to cognition, and the key to its triggering lies in that evocative cognitive event that can promote the understanding of values by people (Thrash and Elliot, 2004). In addition, two important cognitive processing mechanisms, namely, presence and processing fluency, have been found. Firstly, rich, vivid or fluid short video ads can create compelling and authentic experiences for consumers in a virtual environment. This sense of presence greatly stimulates their imagination and inspiration. A respondent illustrates the path through which short videos generate presence and act on customer inspiration: “*Biscuit and power bank videos appeared inauthentic, ‘they are displayed in a showroom and everything is well arranged.’ By contrast, the video about disposable sponge mop is in the home environment, it is an authentic scene, and bring me a sense of reality, as if I were seeing something really happened around. I will be more focused and attracted to watch the video. I really want to have a try*” (Respondent 13). A recent study also confirmed that the key to inspiring consumers with brand video advertising is to allow them to identify important values through alternative experiences (Chang, 2020). Secondly, the vivid and fluent short video ads reduce the difficulty of processing information, and such smooth processing experience is more conducive to understanding the intrinsic value of new things. Similarly, some respondents elaborated on this cognitive processing path: “*These two videos can inspire me because I could understand their intentions, without any difficulty*” (Respondent 21). Based on this, the following proposition is obtained:

P4: Presence, processing fluency, perceived innovation, and perceived convenience establish an important path to connect the informational content characteristic to customer inspiration.

#### Emotional Response

Since emotional content focuses on perceptual description, it mainly arouses the emotion-dominated processing mechanism (Yoo and MacInnis, 2005), i.e., the sensation produced when consumers are exposed to advertising (Macinnis and Jaworski, 1989). The emotional content of short video ads can inspire the emotions of consumers, but only activation pressure and approach motivation (such as curiosity, surprise, and pleasure) can inspire customers. First, some novel short video ads will remind consumers of the existing information gap. A strong desire (curiosity) to obtain the missing information will drive them to search it based on knowledge or information, to strive for the ultimate goal and to spark inspiration. As a respondent said: “*I’m particularly curious about how these amazing and convenient functions of the smart suitcase can be achieved. I’m also curious about how the stickies suck in the dirt. They are quite useful. These two videos make me curious, and I will make it out, see what I can find*” (Respondent 12). In fact, the analysis of grid data suggests that arousing an emotional experience of curiosity is the determinant of identifying potentially high and low inspiring advertisements. Secondly, novel short video ads also create surprise. Therefore, consumers are encouraged to eliminate inconsistencies in their cognitive schema and to try some new things. As one of the respondents mentioned, *“I was amazed at the technological change after watching these two short video ads. That’s quite different from another one. I’d never believed it could be used in this way. And this astonishment aroused my strong interest*” (Respondent 19). This statement is also consistent with the view of Bischof et al. (2020), who believe that surprise has a particularly illuminating quality. Finally, pleasant emotions also stimulate the intrinsic approach motivation of consumers and make them inspired. A respondent mentioned the path in which narrative content evoked the pleasant emotions that finally acted on inspiration experience. “*The content in the first two videos is progressing step by step. I can see the process of production, and imagine what it will be. I feel especially happy when I see the final product. I want to do it myself*” (Respondent 13). Accordingly, the following proposition can be obtained:

P5: Curiosity, surprise, and pleasure form an important path to connect the emotional content characteristic to customer inspiration.

### Influencing Factors of Customer Inspiration in Short Videos

Admittedly, inspiring informational content and emotional content stimulate customer inspiration in most cases, there still exist some factors that can affect the frequency and intensity of customer inspiration in short videos (Thrash and Elliot, 2003, 2004). Research on video advertising have found that only when the risk on product or purchase is high will the informational content positively affect the sharing of advertising (Tellis et al., 2019). Similarly, this study identifies an important personal factor in the formation of short video customer inspiration–personal involvement. Personal involvement includes two constructs, namely, relevance in interest and relevance in demand, both of which meet the criteria to be key constructs. According to the theory of involvement, personal involvement reflects not only the degree of association between consumers and informational content, but that between consumers and emotional content (Zaichkowsky, 1994). Therefore, consumers with high involvement have the strongest response to the inspiring informational content and emotional content, which improves the possibility that customer inspiration will be triggered. On the contrary, consumers with low involvement are impervious to creative new ideas. As one of the respondents said, “*The first two videos are in line with my preferences and interests. I really like the art with lighting effect. I like porphyra rice and I once made it myself. Such content will naturally fill my imagination and make me feel better*” (Respondent 20). Therefore, the following proposition is obtained.

P6: Personal involvement enhances the relationship between content characteristic and customer inspiration.

## Conclusion and Discussion

### Conclusion

With a focus on the source of customer inspiration of short videos and its cognitive psychological process, 25 short video users were interviewed by RGT qualitative research method. Through the analysis of text and grid data, this article constructs the formation path model of short video customer inspiration, and systematically discusses the source, triggering mechanism and influencing factors of short video customer inspiration. The main conclusions are as follows: the inspiring informational content characteristics in short video ads include richness, reliability, vividness, and fluency, while the emotional content characteristics include fun, novelty, and narrativity. However, the commercial content characteristics of short video ads prevent customers from being inspired. Secondly, cognitive processing such as presence, processing fluency, perceived innovation and perceived convenience caused by informational content characteristics and emotional responses such as curiosity, surprise, and pleasure aroused by emotional content characteristics are the internal mechanism that stimulates customer inspiration in short videos. In addition, personal involvement enhances the relationship between inspiring content characteristic and customer inspiration.

### Theoretical Implications

First of all, this article creatively takes customer inspiration as the key construct to explore the effectiveness of short video ads, and provides a new theoretical perspective and methodological instruction for researches and practice on advertising. Specifically, with a different perspective from the previous researches that focused on the key variables such as viewing time (Teixeira et al., 2012), sharing willingness (Berger and Milkman, 2012; Akpinar and Berger, 2017), purchase intention (Lee and Hong, 2016; Liu et al., 2018), sharing behavior (Tellis et al., 2019), sales (Chandy et al., 2001; Guitart and Stremersch, 2021), etc., this article finds that in the current fast-paced and ever-shortening customer journey, customer inspiration helps to explore the importance of short videos in online marketing. Consumers’ attention in short video ads is more effectively converted into a positive purchasing behavior.

Secondly, RGT is applied in this article to sort out the content characteristics that influence the generation of customer inspiration in short videos, the cognitive psychological process triggered by customer inspiration, and the key influencing factors. The findings enrich the existing advertising content feature framework and literature on customer inspiration. In specific, the content feature framework of online video advertising constructed by Tellis et al. (2019) provides detailed instruction on how to design advertisements that drive consumers’ sharing behavior. In spite of the current fad of short video ads, this article further identifies some new information (richness, reliability, vividness, and fluency), and emotional (novelty, fun, and narrativity) content characteristics in the hot trend of short video advertising. It is also found that personal involvement affects the effectiveness of these content features, which, to some extent, supplements the original conceptual framework. Next, responding to the call on contextual interpretation, application (Thrash et al., 2014), and source exploration of inspiration (Liang et al., 2016; Böttger et al., 2017), this article extensively studies the various sources of customer inspiration in short videos, specifies its cognitive process, and developed insights into its formation process.

### Managerial Implications

The research findings may help to resolve some practical problems on how to attract and maintain the attention of consumers and prompt them to make positive response. For online marketers, it is necessary in the design of short videos to have an overall consideration of the inspiring (information, emotion, commerce) content characteristics, increasing richness and reliability of informational content and presenting the information vividly and smoothly. Such practice is conducive to stimulating the cognitive process of customer inspiration. Similarly, emotional content that can arouse consumers positive emotional reactions (curiosity, surprise, pleasure) should also be designed, like adding the funny and novel things to short video ads or displaying presenting the experience of product in a narrative way. In addition, marketers should try to reduce the commercial intention of short-video ads such as sales labels with persuasive bias, thus attracting the attention of consumers to the theme of advertising. Therefore, by increasing the probability of inspiration, consumers can be encouraged to actively participate in short video ads, such as browsing, sharing, purchasing, and other engagement behaviors. The short video platforms can increase the exposure rate of high inspiring short video ads by adjusting the content distribution algorithm so as to attract more consumers for businesses. Furthermore, user portrait algorithm can be improved by collecting data related to user involvement (demands, interests) to increase the frequency and intensity of inspiration, which will greatly improve the rate of purchase conversion of short video ads.

### Limitations and Future Research Directions

As an exploratory study, there inevitably exist insufficiencies that need to be further investigated. Firstly, although the selected samples are representative and the number of samples also meets the requirements of theoretical saturation, the small sample size is not large enough to provide us with sufficient evidence for more convincing conclusions. Future studies should employ a large-scale questionnaire survey to test the reliability of the constructed conceptual model. Second, despite the preciseness of strategies in the selection of short video ads, subjective involvement in the procedure may reduce the validity to some extent. Therefore, attempts can be made to include emotion or sensation analysis of comment text in short video ads in order to sort out the most effective inspiring stimuli for experiment. In addition, the proposed content characteristics (information, emotion, business) that affect the customer inspiration can be well manipulated in the experiment. In future studies, the causal relations in the six research propositions and the impact of each factor on customer inspiration experience should be analyzed and verified by experiments. Finally, while customer inspiration may well explain how short video ads attract attention and motivate positive response, real behavioral indicators are more instructive for marketers and should be applied to the construction of future research framework.

## Data Availability Statement

The raw data supporting the conclusions of this article will be made available by the authors, without undue reservation.

## Ethics Statement

The studies involving human participants were reviewed and approved by School of Economics and Management, Northwest University. Written informed consent for participation was not required for this study in accordance with the national legislation and the institutional requirements.

## Author Contributions

PG: conceptualization, methodology, writing – review and editing, supervision, and funding acquisition. HJ: investigation, resources, and writing – original draft. YX: formal analysis and project administration. YC: data curation and visualization. All authors: contributed to the article and approved the submitted version.

## Conflict of Interest

The authors declare that the research was conducted in the absence of any commercial or financial relationships that could be construed as a potential conflict of interest.

## Publisher’s Note

All claims expressed in this article are solely those of the authors and do not necessarily represent those of their affiliated organizations, or those of the publisher, the editors and the reviewers. Any product that may be evaluated in this article, or claim that may be made by its manufacturer, is not guaranteed or endorsed by the publisher.

## Footnotes

1 Different from the practice of asking respondents to provide interview elements (short video ads), the particularity of customer inspiration requires researchers to guide the selection of elements on the basis of existing theories (Tan and Hunter, 2002). The evocation feature of customer inspiration indicates that it originates from an unconscious thinking or evocative pressure, and is stimulated by new things or new ideas (Thrash and Elliot, 2004). In addition, it is difficult to illustrate the specific sources and characteristics of inspiration (Rauschnabel et al., 2019). As a result, the manipulation of emotions and epiphanies can be realized when the inspiring short video advertisements are selected as external stimuli to the inspiration of the respondents (Oleynick et al., 2014). Before the establishment of the video database, the researchers recruited a wide range of short video ads whose viewers share similar characters to those of the formal respondents. In spite of the similarities of inspirational concept among the respondents (Hart, 1998), the concept of customer inspiration was strictly defined in this study so as to obtain qualified short video ads. In this step, 56 inspirational short video advertisements were collected. These ads were further screened on the principle of inspiring external stimuli (Thrash et al., 2014), namely, (1) whether the selected short video ads are generally persuasive, taking the number of likes, comments, and forwarding as the instructive standard; (2) whether the selected short video ads are endowed with fundamental features of being inspiring; and (3) whether the significant difference in the duration of short video ads would interfere inspiration experience. Through the above steps, a video database composed of 16 short video ads was established (Table 2).
